# *Candida albicans* promotes TSST-1 production by *Staphylococcus aureus* through glucose depletion and relief of CcpA-mediated repression

**DOI:** 10.1128/jb.00427-25

**Published:** 2026-02-05

**Authors:** Mathias Carriou, Cédric Badiou, Alexandre Soulard, Christophe d'Enfert, Karine Dufresne, Gérard Lina

**Affiliations:** 1 CIRI, Centre International de Recherche en Infectiologie, Univ Lyon, Inserm, U1111, Université Claude Bernard Lyon 1, CNRS, UMR5308, ENS de Lyon 236341, Lyon, France; 2 Université Claude Bernard Lyon1, CNRS, INSA Lyon, MAP UMR5240 27098, Villeurbanne, France; 3 Institut Pasteur, Université Paris Cité, INRAe USC2019, Unité Biologie et Pathogénicité Fongiques 27058, Paris, France; 4 Département des sciences biologiques, Université du Québec à Montréal 14845, Montréal, Canada; 5 Hospices Civils de Lyon, Hôpital de la Croix Rousse, Centre de Biologie Nord, Institut des Agents Infectieux, Laboratoire de Bactériologie 379398 , Lyon, France; University of Illinois Chicago, Chicago, Illinois, USA

**Keywords:** *Staphylococcus aureus*, *Candida albicans*, menstrual toxic shock syndrome, TSST-1, glucose, CcpA, SaeRS, interactions, vaginal microbiota, carbon catabolite repression

## Abstract

Menstrual toxic shock syndrome (mTSS) is a rare yet life-threatening disease that is caused when the opportunistic pathogenic bacterium *Staphylococcus aureus* releases the toxic shock syndrome toxin 1 (TSST-1) superantigen, which triggers systemic inflammation. The main risk factor for mTSS is prolonged use of intravaginal products, which facilitates *S. aureus* growth and TSST-1 production in menstrual blood. However, some studies suggest that the vaginal microbiota may also play a role in mTSS occurrence. A previous study reported that the presence of *S. aureus* in menstrual fluids was correlated with the simultaneous presence of *Candida* species, particularly *C. albicans*. Here, we assessed the potential involvement of *C. albicans* in the stimulation of TSST-1 production by incubating *S. aureus* strains with *C. albicans* culture supernatants and measuring TSST-1 production. We found that the growth of *C. albicans* depleted the available glucose, thereby alleviating the CcpA-mediated repression of the *tst* gene, which encodes TSST-1, and enabling activation of its expression by SaeRS activity. These results highlight the importance of vaginal microbiota and nutrient availability with regard to the regulation of *S. aureus* virulence in the context of mTSS.

**IMPORTANCE:**

Menstrual toxic shock syndrome (mTSS) is a life-threatening disease caused by toxic shock syndrome toxin 1 (TSST-1)-producing strains of *Staphylococcus aureus*. While tampons are a known risk factor, the vaginal microbiota may also increase mTSS risk. Our findings reveal that the yeast *Candida albicans*, a frequent colonizer of the vaginal mucosa, stimulates TSST-1 production of *S. aureus* by depleting glucose, a key regulator of *tst* gene expression. This study highlights how *C. albicans*, which is part of the vaginal microbiota, can amplify *S. aureus* virulence through metabolic interactions. These findings may also carry clinical implications by identifying vaginal colonization with *C. albicans* as a potential biomarker for heightened mTSS susceptibility, specifically in individuals harboring TSST-1-producing strains of *S. aureus*.

## INTRODUCTION

*Staphylococcus aureus* (*S. aureus*, SA) is a commensal bacterium found in 30% of the human population, which mainly colonizes the skin and nares and is detectable in the vaginal tract of 10% of healthy women (1, 2). Some *S. aureus* strains produce superantigen toxic shock syndrome toxin 1 (TSST-1), and their presence in the vulvovaginal environment is a major risk factor for menstrual toxic shock syndrome (mTSS). This rare yet severe condition occurs with an estimated incidence of 0.7 per 100,000 menstruating individuals and is characterized by symptoms including high fever, hypotension, macular erythroderma, and multisystem dysfunction (3–5). During menses, blood-soaked intravaginal menstrual products—such as tampons and cups—concentrate nutrients and oxygen in the vaginal niche, potentially promoting colonization and growth of *S. aureus* and enabling TSST-1 production (6, 7). TSST-1 can reach the bloodstream and force polyclonal activation of T cells, leading to massive proinflammatory cytokine release and causing severe mTSS symptoms (8).

TSST-1 is encoded by the *tst* gene, and its production is controlled by various regulators. Catabolite control protein A (CcpA) is a dominant glucose-dependent transcription repressor of the *tst* gene and is associated with carbon catabolite repression (CCR) (9, 10). Another major *tst* regulator is the SaeRS two-component system, encoded by the *S. aureus* exotoxin expression (*sae*) operon, which is reportedly required for *tst* expression (11)*.* Additionally, the quorum-sensing accessory gene regulator (Agr) system positively regulates *tst* by activating transcription of the regulatory RNA RNAIII, which inhibits translation of the repressor of toxins (Rot) messenger RNA, thereby relieving *tst* repression (12, 13)*.* Furthermore, the staphylococcal respiratory response (Srr) system can either enhance or inhibit *tst* transcription under high or low oxygen conditions, respectively (14, 15). Finally, the staphylococcal accessory regulator SarA exhibits nuanced control—inhibiting *tst* expression through direct binding to the *tst* promoter and activating RNAIII transcription (16)*.*

To better prevent mTSS development, it is important to understand the associated risk factors, which may include the vaginal microbiota composition. Numerous studies report that *Lactobacillus* species—mainly *L. crispatus*, *L. gasseri*, and *L. jensenii—*play a protective role in healthy vaginal environments, notably by releasing lactic acid, which lowers the vaginal pH and thereby prevents pathogen proliferation (17–19). Studies have also examined how species within the vaginal microbiota can influence TSST-1 production by *S. aureus*, revealing that *Lactobacillus iners* and *Gardnerella vaginalis* may exacerbate toxin production and immune activation (20, 21). Despite results suggesting the potential importance of fungi in shaping the vaginal microbiota, little is presently known about molecular interactions between fungi and *S. aureus* in the vaginal niche (22–24). Jacquemond et al*.* previously demonstrated that the presence of *S. aureus* during menstruation showed strong positive associations with the presence of *Globicatella*, *Prevotella*, *Gardnerella*, and *Candida* species (25). Notably, *Candida albicans* (*C. albicans*, CA) comprises an average of 60% of *Candida* species and overall fungal populations found in the vaginal tract (26).

In the present study, we explored the role of *C. albicans* in the molecular regulation of TSST-1 production by *S. aureus* and, consequently, in mTSS development. We used *C. albicans* with mutations affecting glucose utilization, media with varying glucose levels, and mutant strains of *S. aureus* to assess the importance of glucose availability with regard to microbial interactions and TSST-1 production. Our results demonstrated that the growth of *C. albicans* depleted glucose levels in the medium, which lifted the CcpA-mediated repression of the *tst* gene and thereby enabled a two- to fivefold increase of TSST-1 production by *S. aureus*, mediated by SaeRS activity.

## RESULTS

### *C. albicans* culture supernatant stimulates TSST-1 production by *S. aureus*

**Fig 1:**
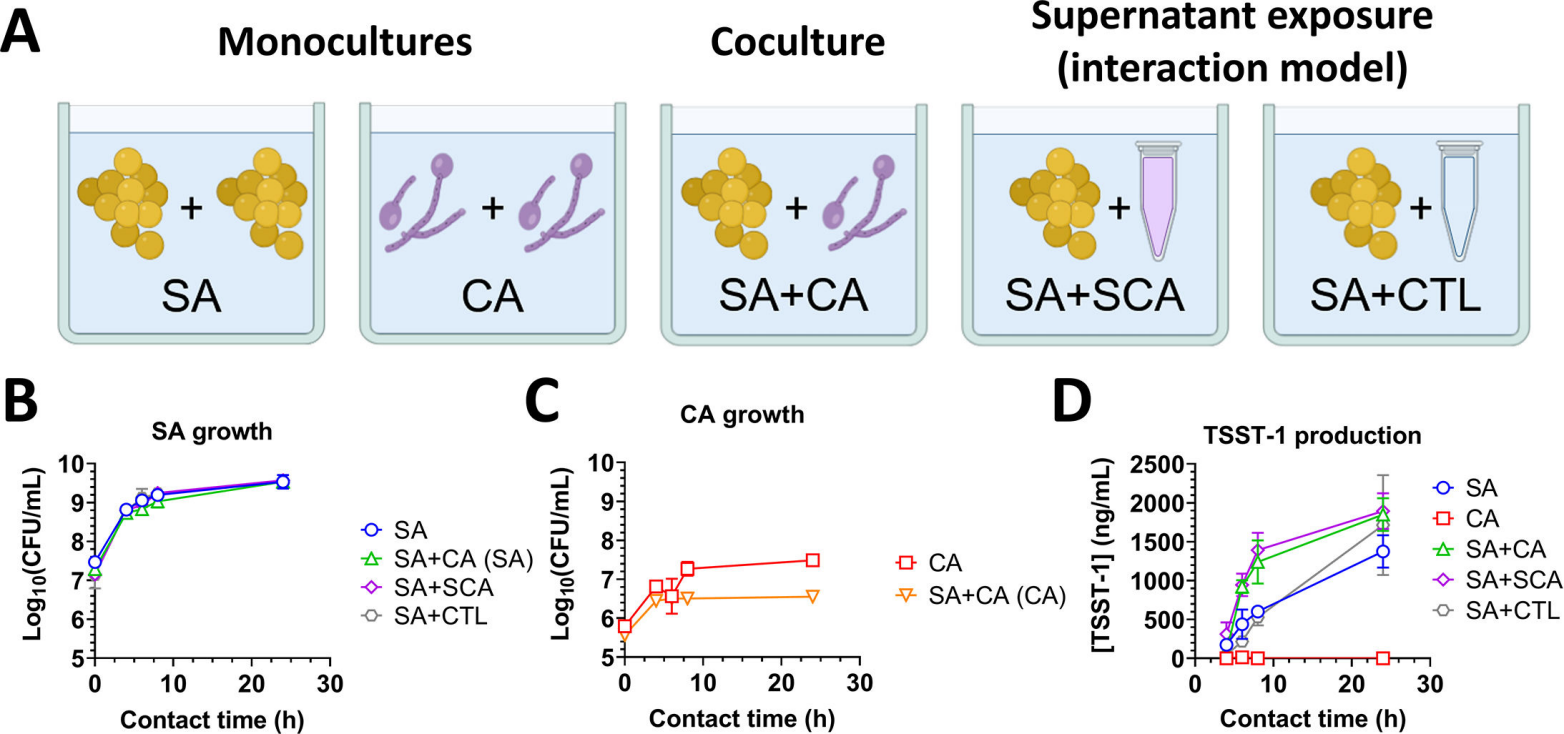
*C. albicans* stimulates TSST-1 production by *S. aureus* during coculture or through supernatant exposure. (**A**) Diagram showing the tested conditions for the interaction model, with *S. aureus* (SA), *C. albicans* (CA), *C. albicans* culture supernatant (SCA), and BHI control medium (CTL). CFU counts of *S. aureus* MN8 (**B**) and *C. albicans* S5314 (**C**) during monocultures, cocultures, or supernatant exposure, after incubation at 37°C for 4 to 24 h. (**D**) TSST-1 production by monocultures of *S. aureus* MN8 and *C. albicans* SC5314 cells, their coculture, and SA with CA supernatant or control BHI medium. *The cumulative data were obtained from three independent experiments.*

To establish the experimental model for studying interactions between *C. albicans* and *S. aureus*, each organism was cultured separately (SA and CA), or co-cultured (SA + CA). *S. aureus* was also incubated in contact with either brain heart infusion (BHI) control medium (SA + CTL) or *C. albicans* culture supernatant obtained after 18 h of culture at 37°C (SA + SCA; Fig. 1A). At t = 0 h, bacterial counts were between 10^7^ and 2 × 10^7^ CFU/mL, while yeast counts were between 3 × 10^5^ and 4 × 10^5^ CFU/mL. This difference in cell counts is likely due to the different cell volumes. Yeast cells are 4–5 times larger than cocci; thus, achieving an equivalent biomass (initial OD_600nm_ of 0.1) will require more bacterial cells than yeast cells (27).

Under all conditions, *C. albicans* and *S. aureus* showed typical growth curves, reaching the stationary phase at 8 h post-inoculation (Fig. 1B). *S. aureus* growth did not significantly differ when incubated alone (SA), versus in coculture with *C. albicans* (SA+CA), incubated with *C. albicans* supernatant (SA+SCA), or with control medium (SA+CTL). *C. albicans* growth was slightly impaired by 4 h in coculture with *S. aureus* (SA+CA), compared to in monoculture (CA), which might result from earlier nutritional depletion of the medium (Fig. 1C). Interestingly, TSST-1 production by *S. aureus* was significantly increased after 6 and 8 h of contact with *C. albicans* (SA+CA) or with its supernatant (SA+SCA), compared to *S. aureus* incubated alone (SA) or with control medium (SA+CTL). TSST-1 levels produced by *S. aureus* did not significantly differ between coculture with *C. albicans* (SA+CA) or exposure to fungal supernatants (SA+SCA) for 6, 8, and 24 h (Fig. 1D). These results indicated that, in this interaction model, *C. albicans* did not interfere with *S. aureus* growth. Moreover, both *C. albicans* itself and its culture supernatant significantly and similarly stimulated TSST-1 production by *S. aureus*. This effect of *C. albicans* on TSST-1 production was likely mediated through an alteration of the medium by the yeast, rather than by direct interaction between the yeast and the bacterium.

### Stimulation of TSST-1 production is mediated by heat-resistant low-molecular-weight molecules and does not depend on pH variations

**Fig 2:**
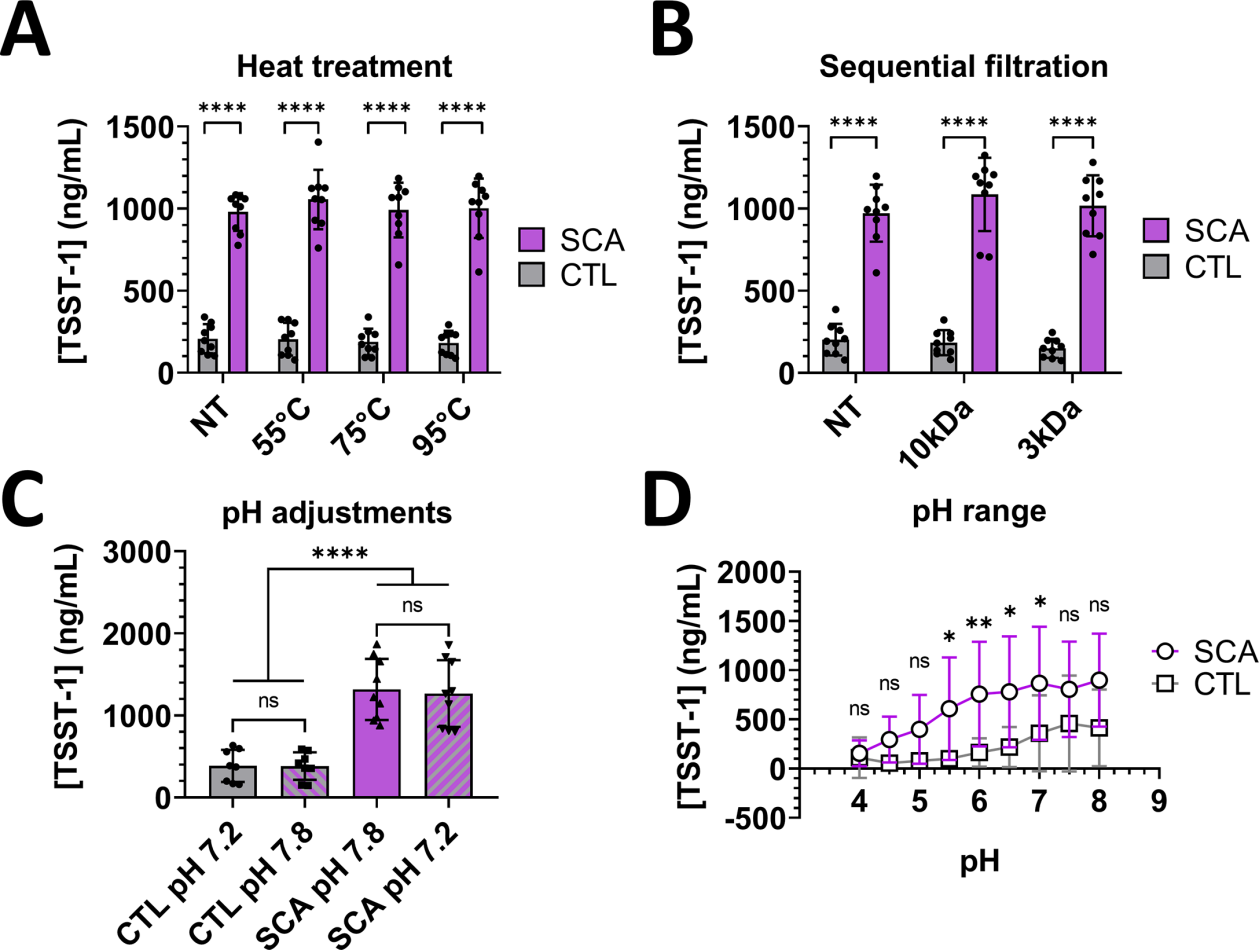
Characterization of the stimulatory activity of *C. albicans* culture supernatant on TSST-1 production by *S. aureus*. TSST-1 production by *S. aureus* MN8 after 6 h of incubation with either *C. albicans* supernatant (SCA, purple bars, circles) or control BHI medium (CTL, gray bars, squares) following heat treatment (**A**), sequential filtration (**B**), or pH buffering (**C, D**). Statistical significance was tested by two-way analysis of variance and post-hoc Dunnett’s test (**A, B, D**), and Kruskal-Wallis test with post-hoc Dunnett’s test (**C**). *P < 0.05; **P < 0.001; ****P < 0.0001. The cumulative data were collected from three independent experiments. ns, not significant.

To assess whether the stimulatory activity of *C. albicans* supernatant was mediated by heat-sensitive substances, we collected fungal supernatant after 18 h of culture at 37°C and heat treated these samples, along with control BHI medium, at various temperatures for 30 min. We then exposed *S. aureus* suspensions to the heat-treated *C. albicans* supernatant or control BHI medium and evaluated TSST-1 production. None of the heat treatments significantly altered the ability of *C. albicans* supernatant to stimulate TSST-1 production, and heat treatment of BHI medium did not affect basal TSST-1 production (Fig. 2A).

Next, we aimed to identify the molecular weight of the molecular mediators of TSST-1 production stimulation. *C. albicans* supernatant obtained from 18 h cultures at 37°C, and control BHI medium, was sequentially filtered to cut-offs of 10 kDa and 3 kDa, and then incubated with *S. aureus* and TSST1 production was quantified. The molecular weight cut-off showed no significant effect, either in comparisons of each supernatant with the corresponding control, or in comparisons among the supernatants or controls (Fig. 2B). Overall, these results demonstrated that the stimulation of TSST-1 production likely occurred through the accumulation or depletion of low-molecular-weight, heat-resistant peptides, compounds, or ions.

Low vaginal pH prevents pathogen proliferation; an increase in vaginal pH can drastically alter vaginal health (18). To assess the role of pH variations in the modulation of TSST-1 production, we adjusted the pH of supernatants from 18 h *C. albicans* cultures to that of the base BHI medium (pH = 7.2; 7.16 ± 0.05), and the pH of base BHI medium was buffered to that of 18 h culture supernatant of *C. albicans* (pH = 7.8; 7.63 ± 0.27)*.* Fungal supernatant and control BHI medium were also adjusted from pH 4.0 (a healthy vaginal pH) to pH 8.0. Basal TSST-1 production did not significantly differ between control BHI media at pH 7.2 versus pH 7.8. Similarly, stimulation of TSST-1 production was not significantly affected by lowering the fungal supernatant pH to 7.2, compared to untreated supernatant at pH 7.8 (Fig. 2C). *C. albicans* supernatants significantly stimulated TSST-1 production only when buffered to pH values between 5.5 and 7. Additionally, TSST-1 production may have been stimulated, although to a non-significant degree, by supernatants of pH ranging from 4.5 to 8.0. TSST-1 production from *S. aureus* was higher when incubated with *C. albicans* supernatants at pH 6 to 8, compared to supernatants at pH 4 and 4.5. No significant difference in TSST-1 production was observed when comparing control medium at all tested pH values (Fig. 2D). Despite the high variability, these results indicated that pH itself was likely not directly responsible for stimulation of TSST-1 production by the yeast but did interfere with basal TSST-1 production. To summarize these first results, TSST-1 stimulation of *S. aureus* by *C. albicans* supernatants appeared to be mediated through the accumulation or depletion of low-molecular-weight, heat-resistant molecules, independently of pH modulation by *C. albicans.*

### Stimulation of TSST-1 production correlates with glucose depletion in medium

**Fig 3:**
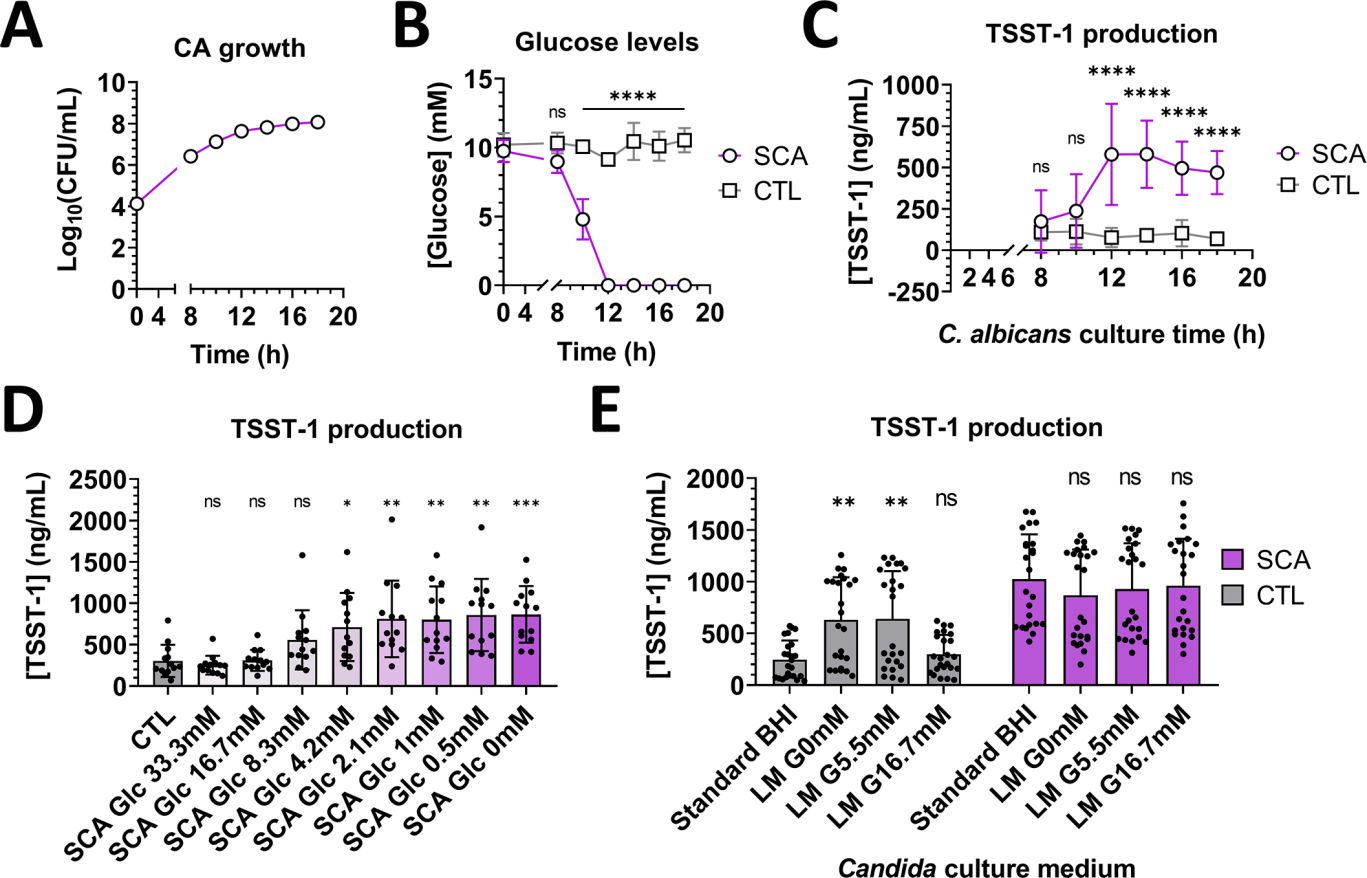
Glucose levels control TSST-1 production in media. (**A**) Concentration of CFUs in cultures of *C. albicans* SC5314 (CA) incubated at 37°C from 0 to 18 h. (**B**) Glucose levels measured in control BHI medium (CTL) and *C. albicans* culture supernatant (SCA) obtained from cultures shown in (**A**). (**C**) TSST-1 production by *S. aureus* MN8 after 6 h of incubation with control BHI medium and *C. albicans* culture supernatant obtained from (**A**). (**D**) TSST-1 production by *S. aureus* MN8 after 6 h of incubation with glucose-supplemented *C. albicans* supernatants and control standard BHI (CTL). (**E**) TSST-1 production by *S. aureus* MN8 after 6 h of incubation with *C. albicans* supernatants obtained from 18 h cultures of *C. albicans* SC5314 in standard commercial BHI or laboratory-made BHI with either 0 mM (LM-G0), 5.5 mM (LM-G5.5mM), or 16.7 mM (LM-G16.7mM) of glucose. Statistical significance was tested using Kruskal-Wallis tests, followed by Dunn’s post-hoc test (**D**) and two-way analysis of variance with post-hoc Tukey’s tests (**B, C, and E**). *P < 0.05; **P < 0.001; ***P < 0.0005. Cumulative data were obtained from three independent experiments. ns, not significant.

Since TSST-1 production is controlled by glucose levels, we next investigated whether the stimulation of TSST-1 production was driven by *C. albicans* glucose consumption. *C. albicans* strain SC5314 WT was cultivated in BHI medium at 37°C for up to 18 h, and samples were collected at different time points for cell counting. Fungal supernatants were harvested and incubated for 6 h with *S. aureus*, followed by titration of TSST-1 production. Starting from an inoculum of 10^4^ CFU/mL, *C. albicans* showed standard growth curves for both biomass and viable cell counts, reaching stationary phase after around 12 h at 37°C (Fig. 3A). Concomitantly, the glucose levels in the fungal supernatant significantly decreased from 10 h of culture and fell to undetectable levels after 12 h of culture (Fig. 3B). TSST-1 production by *S. aureus* significantly increased after incubation with the 12 h culture supernatant of *C. albicans* (Fig. 3C). This suggested that the *C. albicans*-mediated stimulation of TSST-1 production by *S. aureus* was likely correlated with fungal growth phase and the glucose levels in the medium.

To confirm the effect of glucose on TSST-1 production in our interaction model, supernatants obtained from 18 h cultures of *C. albicans* were gradually supplemented with glucose from 0 mM to 33.3 mM (twice the initial glucose concentration of standard BHI medium) and filter-sterilized before the TSST-1 production stimulation assay. TSST-1 production was significantly modulated after contact with supernatants with glucose supplementation ranging from 0 mM to 4.2 mM. Supernatants with higher glucose supplementation were associated with low TSST-1 production, which did not significantly differ from the TSST-1 production obtained after incubation with control BHI medium (Fig. 3D).

**Fig 4:**
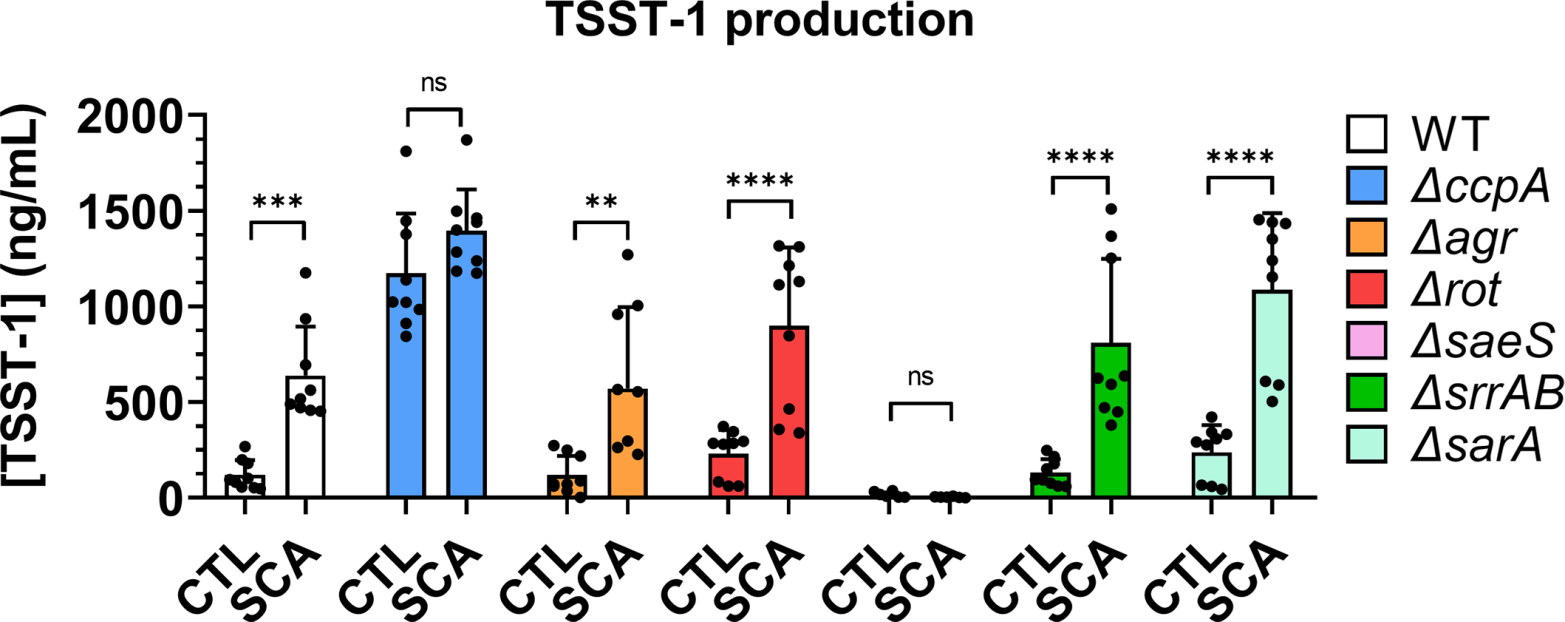
TSST-1 production by *S. aureus* strains lacking regulators of *tst* expression. TSST-1 production by *S. aureus* MN8 WT (white bars), *ΔccpA* (blue bars), *Δagr* (orange bars), *Δrot* (red bars), *ΔsaeS* (pink bars), *ΔsrrAB* (green bars), and *ΔsarA* (teal bars) after 6 h of incubation with 18 h *C. albicans* culture supernatant (SCA) or control BHI medium (CTL). Statistical significance was tested by two-way analysis of variance with post-hoc Tukey’s tests. *P < 0.05; **P < 0.001; ***P < 0.0005; ****P < 0.0001. Cumulative data were obtained from three independent experiments. ns, not significant.

Although glucose levels in *C. albicans* supernatants clearly affected TSST-1 production by *S. aureus*, it could not be excluded that other molecules accumulated or depleted in supernatants might be involved in TSST-1 production. Therefore, we conducted further tests to confirm whether glucose was the sole regulator of the stimulation of TSST-1 production in our interaction model. Laboratory-made (LM) BHI with glucose supplementation at 0 mM (LM-G0mM), 5.5 mM (LM-G5.5mM), and 16.7 mM (LM-G16.7mM) was used for *C. albicans* growth for 18 h, and the supernatant effects on *S. aureus* were investigated in comparison to standard commercial BHI (base glucose concentration of 16.7 mM). The basal TSST-1 production with control LM-G0 and LM-G5.5mM BHI media was significantly higher compared to standard and LM-G16.7mM BHI. Stimulation of TSST-1 production by *S. aureus* was significantly higher than controls only for supernatants from *C. albicans* grown in standard, LM-G5.5mM, and LM-G16.7mM BHI. Supernatants of yeast grown in glucose-free LM BHI exhibited no significant effect on TSST-1 production compared to the controls (Fig. 3E). Overall, these results indicated that, in the present interaction model between *C. albicans* and *S. aureus*, *C. albicans* supernatant stimulated TSST-1 production by *S. aureus* mainly due to glucose levels.

### CcpA and SaeRS control TSST-1 production by *S. aureus* during exposure to *C. albicans* culture supernatant

In *S. aureus*, glucose-mediated repression of *tst* expression is controlled by CcpA (9, 10). To confirm the CcpA-controlled glucose-mediated repression of TSST-1 production, and to further study the regulation of the *tst* gene in this interaction model, *S. aureus* strains lacking the *ccpA* gene or other known regulators of *tst* expression—namely *agr*, *rot*, *saeS*, *srr*, and *sarA*—were incubated with *C. albicans* supernatant or control standard BHI medium. Only the *ΔccpA* and *ΔsaeRS* mutants displayed TSST-1 production profiles different from the WT profile. Specifically, *ΔccpA* showed high basal TSST-1 production, both with *C. albicans* supernatants and control BHI medium, whereas the *ΔsaeRS* strain showed low to no TSST-1 production. The *Δagr*, *Δrot*, and *Δsrr* strains displayed basal TSST-1 production similar to WT. Additionally, *ΔsarA* produced a profile similar to the WT strain, although TSST-1 production after contact with SCA was significantly higher for the *ΔsarA* strain compared to the WT strain (Fig. 4).

### Stimulation of TSST-1 production by *C. albicans* involves hexokinase activity of fungal Hxk2p

**Fig 5:**
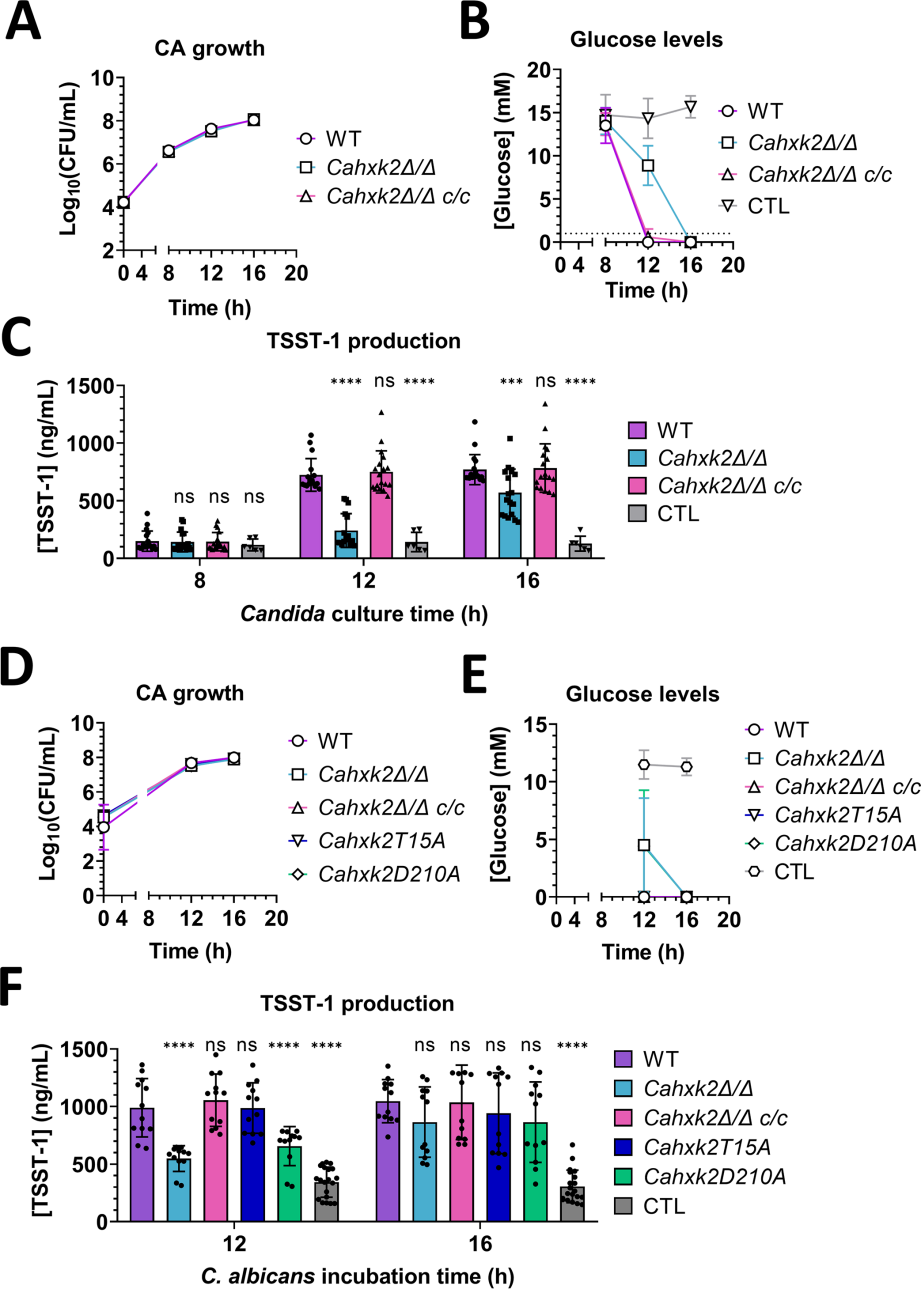
Glucose depletion and fungal hexokinase Hxk2p involvement in the *C. albicans* (CA)-mediated stimulation of TSST-1 production by *S. aureus*. (**A and D**) Concentration of CFUs and (**B and E**) glucose levels measured in cultures of *C. albicans* SC5314 WT (purple, circles), *Cahxk2Δ/Δ* (light blue, squares), *Cahxk2Δ/Δ c/c* (pink, right-side-up triangles), *Cahxk2Δ/Δ T15A* (dark blue, upside-down triangles), and *Cahxk2Δ/Δ D210A* (green, rhombus) at 37°C from 8 to 16 h, and control BHI medium (gray, hexagon). (**C and F**) TSST-1 production by *S. aureus* MN8 after 6 h of incubation with supernatants from WT and mutant strains of *C. albicans*, and control BHI medium. Statistical significance was tested using two-way analysis of variance and post-hoc Tukey’s tests. *P < 0.05; **P < 0.001; ****P < 0.0001. Cumulative data were collected from at least two independent experiments for each graph. ns, not significant.

WT and slow-glucose-depleting mutant strains of *C. albicans* were used to confirm that glucose depletion was due to *C. albicans* growth and metabolism. The *HXK2* gene encodes the hexokinase Hxk2p, which is one of the three glucose-phosphorylating enzymes in *C. albicans* that ensures glucose import and phosphorylation, for the first step of glycolysis. Deletion of both *HXK2* alleles leads to slower glucose depletion in culture medium (28, 29). WT, *Cahxk2Δ/Δ*, and complemented (*Cahxk2Δ/Δ c/c)* strains were cultivated, their CFU counts determined, and their supernatants collected for a TSST-1 production stimulation assay. The three strains displayed similar growth over time (Fig. 5A). After 12 h, the remaining glucose concentration was significantly higher with the *Cahxk2Δ/Δ* strain, compared to the WT and complemented strains. At 16 h of culture, the glucose concentrations were undetectable for all three strains (Fig. 5B). TSST-1 production by *S. aureus* was negatively correlated with the glucose levels in supernatants. Indeed, *S. aureus* produced significantly less TSST-1 during exposure to 12 h *Cahxk2Δ/Δ* supernatants, compared to exposure to 12 h supernatants of WT and complemented strains. However, the 16 h culture supernatants of all three strains increased TSST-1 production by *S. aureus* to the same levels (Fig. 5C).

In addition to its enzymatic activity, Hxk2p is a transcriptional regulator controlling the expression of glucose-repressed genes, associated with the CCR of *C. albicans* (29, 30). To assess which function was affecting TSST-1 production stimulation, yeast mutant strains lacking either regulatory (*Cahxk2T15A*) or catalytic (*Cahxk2D210A*) activity of Hxp2p were cultivated, followed by CFU counts, supernatant recovery, and use in a TSST-1 stimulation assay. At 12 and 16 h, the *Cahxk2D210A* strain showed growth and glucose depletion profiles similar to those of the *Cahxk2Δ/Δ* strain. In contrast, the *Cahxk2T15A* strain displayed 12 h and 16 h growth and glucose depletion profiles similar to those of the WT and *Cahxk2Δ/Δ c/c* strains (Fig. 5D and E). At 12 h of culture, culture supernatants of the *Cahxk2Δ/Δ* and *Cahxk2D210A* strains failed to stimulate TSST-1 production to the same degree as supernatants obtained from the WT, complemented, and *Cahxk2T15A* strains (Fig. 5F). This difference between strains was lost at 16 h of cultures, at which all five strains stimulated TSST-1 production to similar levels. These findings confirmed that *C. albicans* glucose metabolism led to the depletion of glucose in medium, and this process partially relied on the enzymatic function of Hxk2p. Moreover, it was this depletion that elevated TSST-1 production by *S. aureus*, according to our interaction model.

Overall, based on our interaction model of *S. aureus* with *C. albicans* culture supernatants, we showed that TSST-1 production was increased two- to fivefold, and this stimulation was mainly dependent on glucose levels in the medium. *C. albicans* depletes glucose levels during its growth, enabling early release of CcpA-mediated glucose repression of the *tst* gene, which then allows SaeRS-driven gene expression, thereby promoting TSST-1 production.

## DISCUSSION

**Fig 6:**
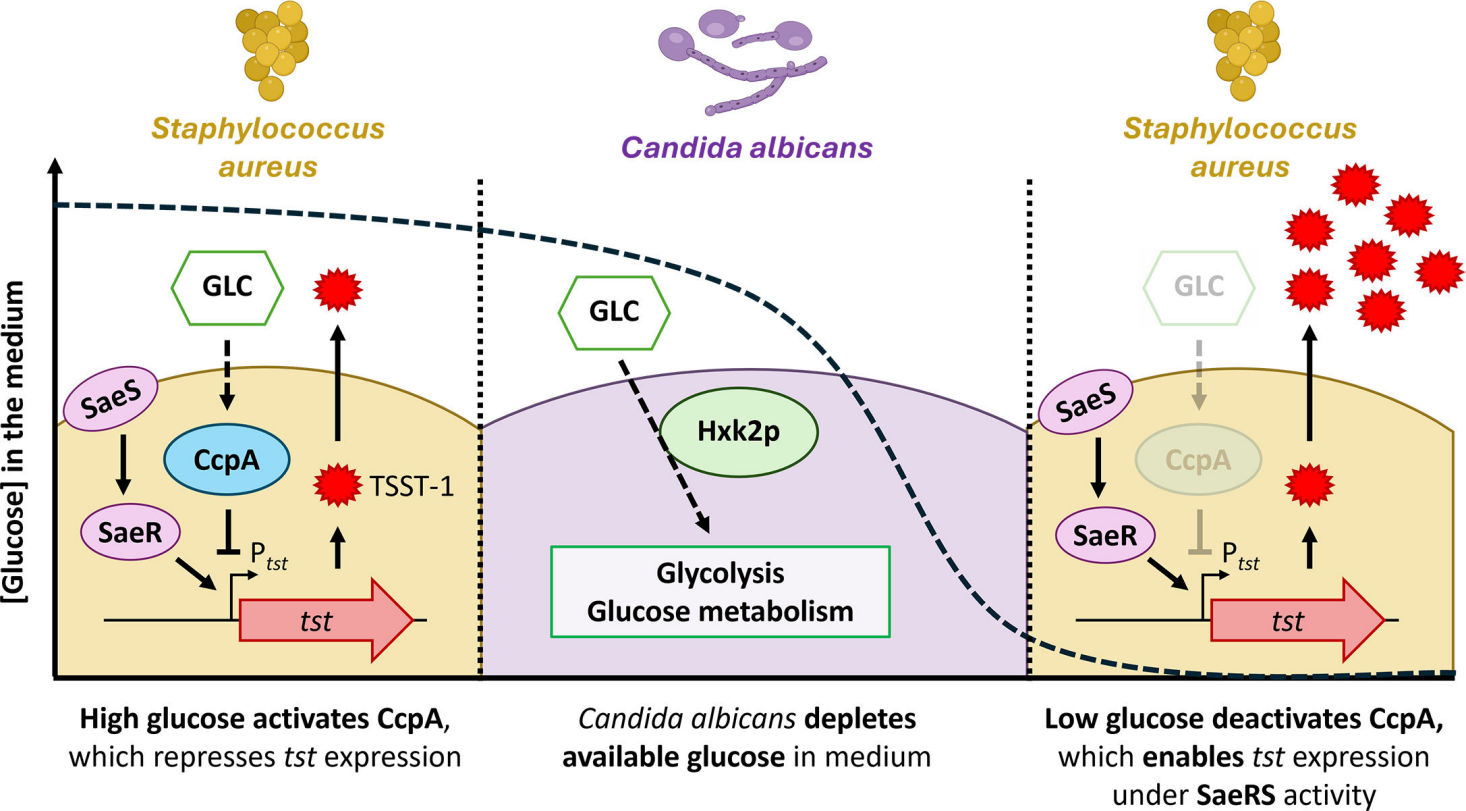
Hypothetical model of the stimulation of TSST-1 production by *S. aureus* through interaction with *C. albicans*. SaeS, *S. aureus* exotoxin expression sensor histidine kinase; SaeR, SaeS-associated response element; Glc, glucose; *tst*, TSST-1-encoding gene; P*_tst_*, *tst* promoter; Hxk2p, hexokinase 2. Solid arrows and dotted arrows indicate direct and indirect action, respectively.

mTSS is a rare severe disease. While awareness about safe use of intravaginal menstrual products has improved, other less controllable factors, such as vaginal microbiota, may affect mTSS risk. In the present study, we demonstrated that the opportunistic fungal pathogen *C. albicans* may promote virulence in *S. aureus*. Specifically, *C. albicans* depletes glucose levels and thereby lifts CCR of the *tst* gene, which is mediated by CcpA, consequently enabling SaeRS-driven transcription of *tst* (Fig. 6).

In our study, glucose depletion was identified as the main signal stimulating TSST-1 production in the interaction between *C. albicans* and *S. aureus*. Glucose levels in cervicovaginal mucus vary over the menstrual cycle, peaking at around 4.4–8.3 mM during the post-ovulatory phase (31). The average bloodstream glucose levels are reportedly around 6 mM (32). Exact glucose concentrations from menstrual fluid samples have not yet been reported. Alterations of glucose availability might result in drastic variations of TSST-1 production and higher mTSS risk during menstruation. Vaginal glucose also originates from glycogen, a large and ramified glucose polymer produced by vaginal and cervical epithelial cells (33). Glycogen can be broken down into glucose by the local microbiota, especially *Lactobacillus* species (34–36). Glycogen is crucial for lactic acid production and *Lactobacillus* colonization, but disruptions in *Lactobacillus* abundance can lead to vaginal dysbiosis and pathogen proliferation (37). Glycogen can also be degraded by vaginal pathogens such as *Gardnerella* species and *C. albicans* (38, 39). No reports have described glycogen transport and degradation by *S. aureus* and its potential effects on virulence and metabolism. Thus, there remains a need for additional studies on how the vaginal microbiota metabolizes vaginal nutrients and their effects on the virulence of pathogens, such as *S. aureus*.

Although our present analyses demonstrated that TSST-1 production was mainly affected by glucose levels, we cannot exclude the potential effects of other metabolites, which could include naturally occurring amino acids, amino-acid-derived metabolites, and heat-stable peptides (40, 41). Further studies are needed to explore the whole exometabolome of *C. albicans* in its interaction with *S. aureus* and their effect on TSST-1 production and the overall virulence of *S. aureus.* Furthermore, the molecular composition of menstrual fluids differs from that of BHI medium, despite their similar neutral pH and various nutrient sources (42). While previous studies have used media simulating vaginal and cervical secretions, no defined medium representing menstrual fluid has yet been published (43).

In our interaction model, only CcpA seemed to be significantly involved in the stimulation of TSST-1 production by *C. albicans*, as SaeRS likely activates *tst* expression independently of *C. albicans* and glucose depletion. Deletions of *ccpA* and *saeRS* yielded results consistent with previously published findings that CcpA is a major *tst* repressor, and SaeRS is a dominant activator of *tst* expression (9–11). On the other hand, deletions of *agr* and *rot* did not lead to altered TSST-1 production under our experimental conditions. Agr and Rot are well-described regulators of exotoxin production, including TSST-1. Tuffs et al*.* (13) demonstrated that Rot overexpression led to low levels of *tst* expression, whereas wild-type levels of Rot seemed to enable relatively high *tst* expression in the MN8 strain, compared to a *Δrot* strain (13, 44). Corroborating these results, Dufresne et al*.* showed that *rot* deletion also led to lower *tst* transcription in a medium simulating vaginal fluids (vaginally defined medium, VDM); they also reported no *tst* expression in a *Δagr* strain grown in VDM (45).

Among other described *tst* regulators, the redox-sensitive SrrAB system reportedly regulates *tst* expression according to oxygen levels. SrrAB seems to both inhibit *tst* expression under microaerobic conditions and enhance *tst* expression under aerobic conditions (14, 15). Dufresne et al*.* reported near-complete extinction of *tst* expression under aerobic conditions in the MN8 *ΔsrrAB* strain (45). Intravaginal menstrual products enable oxygen to enter the microaerophilic vaginal environment, such that SrrAB may be deactivated, enabling TSST-1 production (6, 46). Our interaction model was maintained under aerobic conditions, relevant to mTSS; therefore, the absence of SrrAB-mediated repression of *tst* expression is consistent with previous observations related to TSST-1 inhibition under microaerobic conditions. Finally, although previous reports have demonstrated the role of SarA in the direct and indirect regulation of TSST-1 production (16, 44), in our present study, *sarA* deletion did not alter the stimulation of TSST-1 production by *C. albicans* culture supernatants.

Furthermore, we used variants of fungal Hxk2p to confirm that glucose depletion was indeed caused by *C. albicans* glucose consumption and metabolism. Our results show that this process required the enzymatic activity of Hxk2p but not its regulatory activity. In *C.* albicans, Hxk2p has been reported as a co-regulator, controlling the expression of various genes associated with CCR, along with Mig1p and Mig2p, two transcription factors that are activated when glucose levels are high (29, 47). It is possible that the impairment of Hxk2p regulatory activity in our study might not have been sufficient to alter the CCR. Additional studies using *C. albicans* strains harboring deletions of *MIG1*, *MIG2*, and *HXK2* could be useful for better deciphering the possible impact of CCR disturbance on the interaction between *C. albicans* and *S. aureus*.

Previous studies of *C. albicans–S. aureus* interactions have described the stimulation of alpha-toxin (or alpha-hemolysin) production by *S. aureus* during coculture with *C. albicans* (48, 49)*.* Another study revealed the importance of ribose during this interaction, which is depleted by *C. albicans* during coculture and enables Agr activation through unclear molecular mechanisms (50). The available literature does not include any report of the ribose concentration within the vaginal mucosa, further highlighting the importance of additional studies on the precise metabolite composition of vaginal and menstrual fluids. Notably, in our interaction model, pH alkalinization, *agr*, and *rot* were not necessary for the stimulation of TSST-1 production, which may hint at the involvement of different molecular mechanisms.

In the present study, we only examined the effect of *C. albicans* on *S. aureus*, and not the other way around, since the investigation was focused on TSST-1 production and mTSS. Moreover, this study was specifically designed to focus on the effects of *C. albicans* culture supernatants on TSST-1 production by *S. aureus*. Although TSST-1 production did not differ between this model and cocultures, the interaction between the two organisms might have involved other mechanisms based on molecular crosstalk. Furthermore, we only studied the interaction between *C. albicans* and *S. aureus* since *C. albicans* comprises most of *Candida* species and the overall fungal population of the vaginal microbiota (26, 51). However, the decrease in glucose levels in menstrual secretions is probably not specific to *C. albicans*, and it is highly likely that other microorganisms in the vaginal microbiota can also decrease glucose levels in the presence of *S. aureus* and induce stimulation of TSST-1 production. MacPhee et al*.* (20) demonstrated that supernatants of *Streptococcus agalactiae* culture in BHI significantly increased *tst* expression, while supernatants of *Gardnerella vaginalis* and *Lactobacillus iners* cultures decreased *tst* expression (20). Maduta et al. (2024) showed that supernatants of *Lactobacillus iners* and *Gardnerella vaginalis* cultures in VDM were able to increase TSST-1 production by the MN8 *ΔccpA* strain and enhance T-cell activation in both WT and MN8 *ΔccpA* strains (21). These studies did not investigate glucose levels in the microbial culture supernatants. Despite the inconsistencies, which are likely due to the use of different media, both studies underscore the complexity of microbial interactions in the vaginal ecosystem, particularly related to TSST-1 production, metabolite availability, including glucose, and mTSS development. Additionally, no published report describes an association between mTSS development and the presence of *Candida* species within the vaginal microbiota. Given the results of previous studies, it is likely that mTSS onset and development may be associated with the specific composition of vaginal microbiota that can be isolated from menstrual fluids, including *C. albicans*. Since *C. albicans* represents around 65% of the total fungal vaginal population, and due to its efficient uptake of nutrients, especially glucose, it might quickly colonize and proliferate within menstrual blood, alongside *S. aureus* (26, 52, 53)*.* This could mean that *C. albicans* could expedite glucose depletion, leading to early relief of CcpA-mediated repression of *tst* expression in *S. aureus* and thereby enabling the production of more TSST-1 and triggering mTSS.

In conclusion, here we demonstrated that supernatants from *C. albicans* cultures could stimulate TSST-1 production by *S. aureus*. This occurred because *C. albicans* growth depleted the glucose levels in media, which relieved repression of *tst* gene expression by CcpA and enabled SaeRS-mediated transcription of *tst*. These findings highlight the significance of metabolic interplay within the vaginal microbiota in disease development. Given the central role of TSST-1 in mTSS, our present findings underscore the likely importance of metabolic interactions within the vaginal microbiota in the development of this disease. This study also highlights the broader conceptual intersection of microbial metabolism and virulence, suggesting that metabolic interactions could be a pivotal parameter affecting the onset and severity of polymicrobial infections.

## MATERIALS AND METHODS

### Strains and media

Table 1 lists all strains used in the study. Strains were streaked onto plates of Columbia Agar containing 5% sheep blood (bioMérieux, France) and incubated for 24 h at 37°C for *S. aureus* and 48 h at 30°C for *C. albicans.* To standardize the physiological state before each experiment, the strains were pre-cultivated in BHI liquid medium (BBL BHI, BD 211059), under agitation at 200 rotations per minute (rpm), for 18 h at 37°C for *S. aureus*, and 24 h at 30°C for *C. albicans*. Dilution-plating counts were realized by plating mono-microbial suspensions on trypticase soy agar (TSA; BD). Polymicrobial suspensions were plated on Sabouraud agar (Bacto tryptone 5 g/L, Bacto proteose peptone 5 g/L, glucose 40 g/L, and agar 15 g/L) for *C. albicans* isolation or on mannitol salt agar (#236950, BD) for *S. aureus* isolation. LM-BHI was prepared based on the composition of commercial standard BBL BHI (BD, #211059), including BHI solids 6 g/L, peptone from animal tissue (Peptone A) 6 g/L, peptone from pancreatic digest of gelatin (Peptone G) 14.5 g/L, NaCl 5 g/L, and Na_2_HPO_4_ 2.5 g/L. After dissolution of powders and salts by stirring, LM-BHI was filter sterilized using Nalgene Rapid-Flow 75-mm filter units (Thermo Scientific). Next, D-glucose was added into LM-BHI up to 16.7 mM (LM-G16.7mM), the solution was filter sterilized again, and lower glucose concentrations were prepared by diluting LM-G16.7mM with LM-G0mM.

**TABLE 1:** List of microbial species used in this study

Strain name	Description	References
*Candida albicans*		
SC5314 WT	Wild-type reference strain	54
SC5314 *Cahxk2Δ/Δ*	SC5314 with deletion of both *Cahxk2* alleles	28, 29
SC5314 *Cahxk2* c/c	SC5314 *Cahxk2Δ/Δ* with reintroduction of both *Cahxk2* alleles	28, 29
SC5314 *Cahxk2Δ/Δ* T15A	SC5314 *Cahxk2Δ/Δ* with reintroduction of a *Cahxk2* allele containing the T15A mutation	28, 29
SC5314 *Cahxk2Δ/Δ* D210A	SC5314 *Cahxk2Δ/Δ* with reintroduction of an *HXK2* allele containing the D210A mutation	28, 29
*Staphylococcus aureus*		
MN8	TSST-1^+^ reference strain, isolated from a patient with TSS in the USA in 1980; USA200, CC30	55
MN8 *ΔccpA*	MN8 with deletion of *ccpA*	10
MN8 *Δagr::tet*	MN8 with the *agr* operon replaced by a *tet* tetracycline resistance marker	56
MN8 *Δrot*	MN8 with deletion of *rot*	13
MN8 *ΔsaeS*	MN8 with deletion of *saeS*	11
MN8 *ΔsarA*	MN8 with deletion of *sarA*	11
MN8 *ΔsrrAB*	MN8 with deletion of *srrAB*	15

### *C. albicans* culture supernatant, treatments, and glucose titration

Overnight pre-cultures of *C. albicans* strains were grown in 5 mL BHI, with incubation at 37°C under agitation in DeepWell24 plates (Axygen), to a final OD_600 nm_ of 0.001 (10^4^ CFU/mL). For experiments using LM-BHI, 2 mL samples were collected from the precultures in standard commercial BHI and centrifuged at 15,000 × *g* for 5 min. Then the supernatants were discarded, and the pellets were resuspended in sterile DPBS 1× (Thermo Fisher). The samples were washed in this manner twice and then adjusted to an OD_600 nm_ of 0.001 in LM-BHI.

Cultures were collected at each time point and for each condition, and 1 mL aliquots were used for cell counting by OD_600 nm_ measurement and dilution plating. Culture supernatants were obtained after centrifugation for 30 min at 2,600 × *g* at 4°C and were stored at −20°C until use. Unless otherwise stated, supernatants were obtained from cultures of the SC5314 WT strain after 18 h of growth at 37°C under shaking (OD_600 nm_ ≈ 4–6; 10^8^ CFU/mL). *C. albicans* supernatants were supplemented with glucose by adding 6 g of D-glucose per liter of supernatant (16.7 mM), followed by filter sterilization. Next, these supplemented supernatants were twofold serially diluted to a final concentration of 0.5 mM (0.09 g/L). For heat treatment, 2 mL aliquots of 18 h culture supernatants were heated to 55°C, 75°C, and 95°C in a dry block for 30 min. These temperatures were chosen to establish the thermal stability of regulatory compounds in the media and supernatants and to help with their identification. For sequential filtration, supernatants were first filtered using Vivaspin centrifugal concentrators (Sartorius) with a 10 kDa molecular weight cut-off, then refiltered with a 3 kDa cut-off. The final filtrates for each cut-off were filter sterilized using 0.22 µm syringe filters (Clearline, Dutscher). For pH adjustments, supernatants and control media were buffered to final pH values using HCl 1 M or NaOH 1 M and then filter sterilized. In media and supernatants, the glucose concentration was measured using a Tyson Bio TB100 glucometer and its compatible bands, according to the user manual. The supplied control solution was used to calibrate the glucometer before each measurement session and before using each new test strip batch. Then, 10 µL drops of each sample were placed onto the strip reactive zone to measure glucose levels within the range 1.1–33.3 mmol/L. Concentrations lower than 1.1 mmol/L were reported as undetectable and were considered equal to 0.

### *S. aureus* exposure to microbial supernatants and cocultures

Overnight pre-cultures of *S. aureus* and *C. albicans* were diluted to an OD_600 nm_ of 0.1 in BHI. Cocultures were established by mixing 100 µL of *S. aureus* suspension with 100 µL of *C. albicans* suspension in 96-well microplates (#351172, Falcon). For supernatant exposure, 100 µL of *S. aureus* suspension was mixed with 100 µL of *C. albicans* supernatants or control medium. For monocultures, *S. aureus* or *C. albicans* suspensions were prepared at a starting OD_600 nm_ of 0.1 in 200 µL. All cultures were incubated at 37°C for 4–24 h, under agitation at 200 rpm. Supernatants from cocultures and fungal supernatant exposures were collected after 30 min of centrifugation at 2,600 × *g* and at 4°C and were stored at −20°C until use.

### Toxin titration by enzyme-linked immunosorbent assay

Toxins produced in contact with supernatants were quantified by sandwich enzyme-linked immunosorbent assay (57). Anti-TSST-1 capture antibodies (monoclonal) and anti-TSST-1 secondary antibodies (F[ab’]2 fractions, polyclonal, biotin-coupled) were obtained from AgroBio. Detection was performed by adding horseradish-peroxidase-coupled streptavidin, 3,3',5,5'-tetramethylbenzidine substrate (Seracare), and then H_2_SO_4_ 1 M was added to stop the reaction. Colorimetric signals were read at λ = 450 nm using a Biorad microplate reader.

### Statistical analysis and figure creation

Statistical analyses were performed, and graphs were constructed using GraphPad PRISM. In the graphs, each dot represents one biological replicate, each originating from one initial isolated colony. Graphs show the mean values for each group, with error bars representing the standard deviation, using cumulative data from several independent experiments. Data were tested for significance using appropriate tests, including the Kruskal-Wallis test and two-way analysis of variance with appropriate post-hoc tests for between-group comparisons. For all tests, a *P* value of <0.05 was considered to indicate statistical significance. Diagrams were created using BioRender and Microsoft PowerPoint.
